# No interaction between serotonin transporter gene (5-HTTLPR) polymorphism and adversity on depression among Japanese children and adolescents

**DOI:** 10.1186/1471-244X-13-134

**Published:** 2013-05-10

**Authors:** Akemi Tomoda, Shota Nishitani, Naomi Matsuura, Takashi X Fujisawa, Junko Kawatani, Daiki Toyohisa, Mai Ono, Kazuyuki Shinohara

**Affiliations:** 1 Research Center for Child Mental Development, University of Fukui, Fukui, Japan; 2 Department of Neurobiology& Behavior Unit of Basic Medical Sciences Course of Medical & Dental Sciences, Nagasaki University Graduate School of Biomedical Sciences, Nagasaki, Japan; 3 School of Education, Tokyo University and Graduate School of Social Welfare, Tokyo, Japan; 4 Department of Child Development, Faculty of Life Sciences, Kumamoto University, Kumamoto, Japan

**Keywords:** Child depression, Gene-by-environment (G × E) interaction, Serotonin transporter gene (*5-HTTLPR)* Polymorphism, Early adversity

## Abstract

**Background:**

Identification of gene × environment interactions (G × E) for depression is a crucial step in ascertaining the mechanisms underpinning the disorder. Earlier studies have indicated strong genetic influences and numerous environmental risk factors. In relation to childhood and adolescent depression, evidence is accumulating that the quality of the parental environment is associated with serotonin biology in children. We hypothesized that maternal depression is a crucial environmental risk factor associated with serotonin-regulating genes.

**Methods:**

This study was designed to ascertain the G × E interaction for diagnosis of depression in a Japanese pediatric sample. DNA samples from 55 pediatric patients with depression and 58 healthy schoolchildren were genotyped for the *5-HTT* (2 short (S) alleles at the *5-HTT* locus) promoter serotonin-transporter-linked polymorphic region (*5-HTTLPR*) polymorphism. We examined whether an adverse parental environment, operationalized as the mother’s history of recurrent major depressive disorder, interacts with *5-HTTLPR* polymorphism to predict patients’ depression symptoms.

**Results:**

Binary logistic regression analyses revealed that maternal depression (adversity), gender, and FSIQ significantly affect the diagnosis of depression among children and adolescents. However, no main effect was found for adversity or genotype. Results of multivariable logistic regression analyses using stepwise procedure have elicited some models with a good fit index, which also suggests no interaction between *5-HTTLPR* and adversity on depression.

**Conclusions:**

To assess G × E interaction, data obtained from children and adolescents who had been carefully diagnosed categorically and data from age-matched controls were analyzed using logistic regression. Despite an equivocal interaction effect, adversity and gender showed significant main effects.

## Background

Depression, an etiologically heterogeneous group of brain disorders characterized by widely various symptoms that reflect altered cognitive, psychomotor, and emotional processes [1,2], strongly affects 3–5% of children and adolescents [3]. Depression negatively impacts growth and development, school performance, and peer or family relationships; it can lead to suicide [4-6]. Biomedical and psychosocial risk factors include a family history of depression, female sex, childhood abuse or neglect, stressful life events, and chronic illness [3,7,8]. Previous results of studies suggest that exposure to childhood adversity is associated with depression symptoms [9-12]. Although people at risk tend to have poor life outcomes, some people do well despite adversity. Protective factors such as good intellectual skills, positive temperament, parental warmth, parental involvement, and strong social connections are believed to play an important role in protecting individuals from poor development, psychological problems, and mental illness from the viewpoint of genetic etiology [13].

Results of previous studies suggest that homozygous carriers of the short allele (*S/S*) of the serotonin transporter gene-linked polymorphic region (*5-HTTLPR*) might be at increased risk for depression if they have also been exposed to early or current adversity or stress [14].

A growing body of evidence suggests that *5-HTTLPR* interacts with adverse environmental influences to produce increased risk for the development of depression, although the underlying mechanisms of this association remain largely unexplored [15-19]. Various reports have described that *5-HTTLP*R genotype moderates the relation between stress and depression and interacts to contribute to risk for depression in children [20-22]. However, recent meta-analyses have produced no evidence that the *5-HTTLPR* genotype alone or in interaction with stressful life events is associated with an elevated risk of depression [23], or that the main effect of *5-HTTLPR* genotype and the interaction effect between *5-HTTLPR* and stressful life events on risk of depression are negligible [24]. Therefore, we should conduct an examination to incorporate consideration of the possibility of other confounding variables such as gender, age, and socioeconomic status along with stressful life events.

Research in this area can recast our thinking about the role of early experience in psychopathology and genetic interaction. Considering previous research, we hypothesized that interaction between *5-HTTLPR* and early adversity is involved in the etiology of childhood depression. No study has tested Japanese children and adolescents. Therefore, the aim of this study is to examine whether opposite G (*5-HTTLPR* genotype) × E (maternal depression) interactions can be confirmed among Japanese children and adolescents. Our additional research findings will contribute to the resolution of inconsistencies in the literature. One principle aim in the current study was to test whether opposite gene-by-environment (G × E) interactions with maternal depression can be found for Japanese children and adolescents with childhood depression. This study examined G × E interactions in a sample of children with Japanese pediatric depression to test the hypothesis that *5-HTTLPR* is involved in the etiology of childhood depression.

## Methods

### Ethics statement

The Committee of Life Ethics, Graduate School of Medicine, Kumamoto University approved the study protocol, Assurance # KUM0313. During the review of this Project, the committee specifically considered (i) the risks and anticipated benefits, if any, to subjects; (ii) the selection of subjects; (iii) the procedures for securing and documenting informed consent; (iv) the safety of subjects; and (v) the privacy of subjects and confidentiality of the data. All potential participants who declined to participate or otherwise did not participate were eligible for treatment (if applicable) and were not disadvantaged in any other way by not participating in the study. According to the Declaration of Helsinki, parents or guardians of all participants provided written informed consent for children to participate in the study after the study procedures had been explained to them.

### Subjects

This study examined 55 unmedicated patients with depression: 21 boys and 34 girls aged 8–16 years (mean age, 14.3 years; standard deviation [SD], 1.9 years) who were referred to our laboratory during 2007–2011 for examination of depression (Table 1). All the patients were referred from their private pediatric clinics. For that reason, there might be referral bias related to severity despite the drug-naïve condition used for this study. All patients satisfied diagnostic criteria for depression of the Diagnostic and Statistical Manual of Mental Disorders, Fourth Edition, Text Revision (DSM-IV-TR) (American Psychiatric Association, 2000) [2]. To exclude other psychiatric diagnoses, the Mini-International Neuropsychiatric Interview for Children and Adolescents (MINI Kids) was administered by a licensed pediatric-psychiatric clinician. The patient group did not have high levels of suicidality or comorbidity.

**Table 1:** Demographics, early adversity, and clinical characteristics in individuals in patients and healthy controls

	**Healthy controls (n = 58)**	**Patients (n = 55)**	***t-test, ANOVA, other t(F)-value***	***P-value***
Demographics				
Age (years)	*13.8 ± 2.1*	*14.3 ± 1.9*	*1.21*	*0.4*
Gender (Males/Females)	*40 M/18 F*	*21 M/34 F*	*Fisher Exact*	*0.01*
Early Adversity	*0*	*11*	*Fisher Exact*	*<0.001*
Socioeconomic Status	*4.1 ± 0.6*	*4.0 ± 0.7*	*2.62*	*0.11*
Clinical Characteristics				
SDS Score*	*36.47 ± 6.19*	*56.95 ± 6.42*	*(−235.96)*	*<0.001*
DSRS-C Score*	*7.51 ± 4.33*	*21.92 ± 6.23*	*(189.03)*	*<0.001*
Full Scale IQ*	*109.56 ± 12.15*	*94.29 ± 16.49*	*−6.43*	*0.01*
Verbal IQ*	*105.74 ± 9.92*	*89.24 ± 17.37*	*−9.44*	*0*
Performance IQ*	*108.74 ± 10.69*	*95.78 ± 15.60*	*7.67*	*0.01*
Verbal Comprehension Index*	*109.12 ± 13.00*	*92.38 ± 22.08*	*9.05*	*0*
Perceptual Organization*	*104.41 ± 11.98*	*88.90 ± 21.67*	*4.2*	*0.04*
Freedom from Distractibility*	*107.35 ± 12.39*	*91.7 ± 22.36*	*3.27*	*0.08*
Processing Speed Index*	*109.06 ± 10.50*	*89.92 ± 25.61*	*7.99*	*0.01*
Youth Self Report Score (total)	*53.9 ± 25.5*	*84.5 ± 35.9*	*23.59*	*<0.001*
Youth Self Report Score (internal)	*8.3 ± 7.2*	*25.1 ± 8.3*	*63.91*	*<0.001*
Youth Self Report Score (external)	*10.2 ± 6.7*	*14.1 ± 8.6*	*5.46*	*0.02*

* ANCOVA with age and gender as covariates. Values are presented as the mean and SD.

DSRS-C, Birleson Depression Self-Rating Scale for Children.

To obtain data from normal age-matched healthy controls (HC), healthy schoolchildren and adolescents aged 8–16 years were recruited as subjects from the community; school students were targeted. The control group comprised 40 boys and 18 girls (mean age, 13.8 years; SD, 2.1 years). None had below-average IQ, physical problems, or psychiatric psychopathology.

All participants’ race/ethnicity was Japanese. Patients who had undergone treatment with antidepressants or hypotension drugs, or who had diagnoses of neurological illness, migraine, obstructive sleep apnea, below average IQ, or severe psychopathology were excluded from the study. Severe psychopathology was evaluated by referral to at least one pediatric psychiatrist if the patient presented indicative symptoms.

No control subject had any history of DSM-IV-TR Axis I Disorder (based on MINI Kids) including attention deficit hyperactivity disorder (ADHD) or other pervasive developmental disorder, or any history of any form of abuse or drug abuse. Table 1 presents clinical characteristics of the subjects.

As an adult control reference, we also used commercially supplied DNA of Japanese adult subjects (age 44.26 ± 13.58, 50 male and 50 female) supplied by the Health Science Research Resources Bank, Osaka, Japan.

### Assessments

#### Depression scales

The severity of depressive symptoms was measured using the Zung self-rating depression scale (SDS) and the Birleson Depression Self-Rating Scale for Children (DSRS-C), which are similar to the children’s depression inventory used in pediatric psychiatry. The validity and reliability of Japanese version of these scales were confirmed [25,26]. Methods used for these analyses were described previously [27].

#### Wechsler Intelligence Scale for Children-Third Edition (WISC-III)

An individually administered measure of intelligence intended for children aged 6 years to 16 years and 11 months, WISC-III, was administered by a licensed pediatric-psychological clinician to estimate intellectual capacity [28]. The version is applicable to Japanese children and adolescents.

#### Child Behavior Checklist Youth Self-Report (CBCL-YSR) and SES

Patients with child depression had mean scores of 25 and 14, respectively, for Internalizing and Externalizing Problems on the CBCL Youth Self-Report [29].

Low income and poverty might be important developmental risk factors related to psychopathology [30]. The parental socioeconomic status (SES) test was administered as a composite measure of socioeconomic status [31,32]. Socioeconomic status was classified as follows: annual income below 2 million yen, 1; 2–4 million yen, 2; 4–6 million yen, 3; between 6 and 8 million yen, 4, 8–10 million yen, 5; 10–12 million yen, 6; more than 12 million yen, 7.

#### Early adversity

The patients’ mothers were also administered a clinical interview by a specialized physician, the Structured Clinical Interview for the DSM-IV (SCID), to assess their Axis-I disorders. They were investigated if they had either (1) no current/past disorder (low early adversity); or (2) at least two major depressive episodes during their children’s lifetime but were currently in remission (high early adversity). Approximately 20% (*n* = 11) of the mothers had recurrent depression; the remaining 80% (*n* = 44) had no history of mental health disorders. No control subject’s mother had high early adversity. Selected from the same community sample, control subjects’ mothers with no history of psychiatric problems or exposure to traumatic events served as a *no early adversity* contrast group.

#### Genotyping

Genomic DNA was extracted directly from blood samples of patients using standard techniques (MagNa pure LC350; Roche Diagnostics Corp.). In control subjects, we used a mouth swab sampling technique and extracted the DNA from buccal cells using a standard commercial extraction kit QIAamp DNA Micro Kit (Qiagen Inc., Tokyo, Japan). This study was conducted with ethnically homogeneous individuals (all were of Japanese descent) [33].

The serotonin transporter gene (*5-HTTLPR*) of the 5-HTT gene regulatory region was amplified by polymerase chain reaction (PCR) with forward primer (5’-GGCGTTGCCGCTCTGAATGC-3’) and reverse primer (5’-GAGGGACTGAGCTGGACAACCAC-3’). For PCR, 10 ng of genomic DNA was used in a 25-μL reaction mixture containing 0.5 U of KOD FX Neo (Toyobo Co. Ltd.) and 10 pmol of each primer in PCR buffer for KOD FX Neo (Toyobo Co. Ltd.). Cycling conditions were the following: denaturation (94°C for 2 min) and 30 cycles of amplification (98°C for 10 s, 63°C for 30 s and 68°C for 30 s). The PCR products were separated using electrophoresis in a 3% agarose gel and visualized by UV after ethidium bromide staining. A 484 bp band was observed for the short (S) allele, and a 528 bp band for the long (L) allele; heterozygous samples showed both the alleles. Two investigators scored allele sizes independently. Any inconsistency was reviewed and procedures were repeated if necessary. Genotyping was conducted blindly without knowledge of the clinical information.

The PCR-based analyses revealed that the distribution of allele frequency in the patients was 76% for the S-allele and 24% for the L-allele (Table 2). The distribution of the S/S, S/L, and L/L genotypes was, respectively, 60% (*n* = 33), 33% (*n* = 18), and 3% (*n* = 7). In the Japanese population, LL type of *5-HTTLPR* has been reported as rare. Therefore, it might be better to examine the *5-HTTLPR* proportion using Fisher’s exact test or MCMC. However, some previous reports have described the use of Hardy–Weinberg equilibrium examination empirically [34,35]. We also administered Hardy–Weinberg equilibrium examination. The observed genotype distribution was similar to the expected Hardy–Weinberg equilibrium distribution. The genotypes were divided into S/S and S/L + L/L genotypes because only four subjects with the L/L genotype were found in our sample. This genotype is uncommon among Japanese people [36].

**Table 2:** 5-HTTLPR genotype distributions and allele frequencies in controls and depressive patients

	**Genotypes, n (%)**			**Allele frequencies, n (%)**	
	**S/S**	**S/L**	**L/L**	**S**	**L**
Patients	*33 (60.0)*	*18 (32.7)*	*4 (7.3)*	*84 (76.4)*	*26 (23.6)*
Age-matched Controls	*32 (62.7)*	*16 (31.4)*	*3 (5.9)*	*80 (78.4)*	*32 (21.6)*
Adult Controls	*51 (51.0)*	*47 (47.0)*	*2 (2.0)*	*149 (74.5)*	*51 (25.5)*

#### Data analysis

The clinical variables were compared in both groups using *t*-tests for quantitative data and Fisher exact tests for qualitative data. Data are expressed as the mean ± SD. To explore the relation between depressive symptoms and the genotypes of the *5-HTTLPR* polymorphism, analysis of covariance (ANCOVA) was performed for each of the depression scores (total SDS and DSRS-C scores) using age and gender as covariates because it has been suggested that a variable such as age might affect responses to the depression rating [30]. To confirm the main effect on the diagnosis of depression, we used binary logistic regression and set genetic, environmental factors, and the effect of the interactions between *5-HTTLPR* genotype and adversity as independent variables.

Next, multivariable logistic regression analyses were performed. The dependent variable was the status of diagnosis (0 = no diagnosis, 1 = diagnosed as depression). All predictor variables were entered simultaneously into the model. Some possible models were conducted using stepwise procedures. Finally, two-way analysis of variance (ANOVA) was conducted to assess the effect of G × E interaction in each genotype group.

All statistical tests (*t*-test, ANCOVA and Fisher exact test, logistic regression analyses, and two-way ANOVA for discrete variables) were two-sided; *P* values less than .05 were inferred as statistically significant. Statistical analyses were conducted using software (SPSS Statistics 20; SPSS Inc., Chicago, IL).

## Results

### Demographics and IQ

Table 1 shows that the patient and control groups were well matched in terms of age (*F* =1.21, *P* = 0.40) and SES (*F* = 2.62, *P* = 0.11). Female predominance was found in the patient group (Fisher exact, *P* = 0.008). Subjects in the patient group were predominantly male (69%), whereas controls were predominantly female (61%; Table 1). Of 55 children and adolescents in the patient group, 11 had early adversity, although none had it in the HC group. As expected, the patient group had high SDS scores with a mean of 57.0 ± 6.4 (*F* = 235.96, *P* < 0.001) and high DSRS-C scores with a mean of 21.9 ± 6.2 (*F* = 189.03, *P* < 0.001). In addition, those who were diagnosed as having depression had a significantly higher total score of CBCL-YSR (*F* =23.59, *P* < 0.001) and internalizing score of that (*F* = 63.91, *P* < 0.001) than those of the HC group.

The patient group had significantly lower full-scale IQ (FSIQ) without discrepancies between verbal IQ (VIQ) and performance IQ (PIQ), compared to the HC group (94.3 ± 16.5 vs. 109.6 ± 12.2) (*F* = 6.43, *P* = 0.013; Table 1). Similarly, VIQ and PIQ showed significant differences in scores between the two groups (VIQ, *F* = 9.44, *P* = 0.003; PIQ, *F* = 7.67, *P* = 0.007).

The genotypic and allelic frequencies of the *5-HTTLPR* variants are presented in Table 2. The distribution of allelic frequency of the patients and age-matched controls was found to be almost identical to that previously reported in Japanese people, exhibiting a similar tendency to that of our Japanese adult sample (frequency for the L/L, S/L, and S/S genotypes was: 7, 33, and 60% in the patients and 6, 31, and 63% in the age-matched controls, respectively). The overall pattern of results derived by genetic studies of *5-HTTLPR* is most suggestive for a dominant mode of action of the S allele [37]. We therefore analyzed S/L heterozygous patients grouped with L/L homozygous patients (i.e., L/L + S/L versus S/S). No significant difference was found in the genotypic or allelic frequencies of *5-HTTLPR* between both groups, suggesting that no sampling bias exists in the study.

As shown Table 1, significant differences were found between two groups in FSIQ, depression scores, and scores of CBCL. Because of the significant main effects of FSIQ and gender, we assumed that interaction effects between variables such as SES × age, SES × FSIQ, and gender × age would be large.

### Evaluation of binary logistic regression analyses

In this study, we examined the main effect of diagnosis as depression in childhood and adolescence using binary logistic regression analyses. Adversity, gender, and FSIQ were found to be significantly associated with depression [adversity, *P* <0.0001, OR = 2129500000; gender, *P* =0.008, OR (95% CI) = 0.345 (0.158–0.754); FSIQ: *P* =0.001, OR (95% CI) =0.931 (00.892–0.971)]. A significant G × E interaction was observed (*P* <0.0001, OR = 1142600000). However no significant main effect in genotype was found (Table 3).

**Table 3:** Binary logistic regression analyses for variables of genotype, adversity, gender, age, SES, FSIQ, and Genotype (G) × Environment (E)

					
**Genotype (G)**					
B	SE	Wald	* p value*	OR	95% CI
* 0.11*	* 0.32*	*0.119*	* 0.730*	* 1.12*	*0.600-2.073*
Adversity (E)					
B	SE	Wald	* p value*	OR	95% CI
* 21.48*	* 12118.64*	*0.000*	* 0.000*	* 2129489594*	*0.000-*
Gender					
B	SE	Wald	* p value*	OR	95% CI
* −1.06*	* 0.4*	* 7.11*	* 0.01*	* 0.35*	*0.158-0.754*
Age					
B	SE	Wald	* p value*	OR	95% CI
* 0.09*	* 0.1*	* 0.73*	* 0.39*	* 1.09*	*0.894-1.332*
SES					
B	SE	Wald	* p value*	OR	95% CI
* −0.5*	* 0.31*	* 2.56*	* 0.110*	* 0.61*	*0.330-1.119*
FSIQ					
B	SE	Wald	* p value*	OR	95% CI
* −0.07*	* 0.02*	* 10.9*	* 0*	* 0.93*	*00.892-0.971*
Gene × Environment(E)					
B	SE	Wald	* p value*	OR	95% CI
* 20.86*	* 13894.65*	*0.000*	* 0.000*	* 1142607181*	*0.000-*

note: B=unstandardized partial regression coefficient, SE = standard error, OR = odds ratio, Wald = (SE/B)2, CI = confidence interval.

Dependent variable is existence of diagnosis (0 = no, 1 = childhood depression).

Independent variables: 5-HTTLPR genotype (0 = s/s, 1 = s/l, 2 = l/l), Adversity (maternal depression: 0 = no, 1 = yes). Gender (0 = female, 1 = male), SES (from 0 = low income to 5 = high income).

### Evaluation of multivariable logistic regression analyses

As results of multivariable logistic regression analyses using stepwise procedure, we confirmed three models that have goodness of fit (Table 4). In Model 1, significant effects of G × E interaction, adversity, gender, and SES were found [fitness index of this model: χ^2^ = 61.216 (*P* <0.001), -2log likelihood = 37.706, discrimination accuracy = 96.9%]. In Model 2, the effect of G × E interaction was excluded and the fitness index of the model was changed [fitness index of this model, χ^2^ = 61.216 (*P* <0.001), -2log likelihood = 37.706, discrimination accuracy = 96.9%]. Finally, Model 3, the simplest, was inferred when all variables were used simultaneously in the analyses. A significant main effect of gender and FSIQ was found [fitness index of this model, χ^2^ = 50.660(*P* <0.001), -2log likelihood = 48.262, discrimination accuracy = 96.9%].

**Table 4:** Multivariable logistic regression analyses using stepwise procedure

						
**Model1**						
	B	SE	Wald	p value	OR	95% CI
G × E interaction	*−1.39*	*25254.67*	*0.000*	*1.000*	*0.25*	*0.000-*
Genotype (G)	*−1.54*	*0.98*	*2.447*	*0.12*	*0.22*	*0.031-1.475*
Adversity (E)	*21.04*	*21970.77*	*0.000*	*1*	*1371627685*	*0.000-*
Gender	*23.7*	*6397.2*	*0.000*	*1*	*0.000*	*0.000-*
Age	*0.1*	*0.234*	*0.18*	*0.67*	*1.1*	*0.697-1.747*
SES	*−1.64*	*0.77*	*0.000*	*1*	*1371627685*	*0.000-*
FSIQ	*−0.79*	*0.39*	*4.057*	*0.04*	*0.92*	*0.855-0.998*
Fit index of this model: χ2 = 61.216 (p < 0.001), -2log likelihood = 37.706, discriminate accuracy = 96.9%						
**Model2**						
	B	SE	Wald	p value	OR	95% CI
Genotype (G)	*−1.54*	*0.98*	*2.447*	*0.12*	*0.22*	*0.031-1.175*
Adversity (E)	*20.21*	*10910.59*	*0.000*	*1*	*599809518*	*0.000-*
Gender	*−23.7*	*6393.91*	*0*	*1*	*0.000*	*0.000-*
Age	*0.1*	*0.23*	*0.18*	*0.67*	*1.1*	*0.697-1.747*
SES	*−1.64*	*0.77*	*4.567*	*0.03*	*0.2*	*0.043-0.873*
FSIQ	*−0.08*	*0.04*	*4.06*	*0.04*	*0.92*	*0.855-0.998*
Fit index of this model: χ2 = 61.216 (p < 0.001), -2log likelihood = 37.706, discriminate accuracy = 96.9%						
**Model3**						
	B	SE	Wald	p value	OR	95% CI
Gender	*−22.31*	*7045.95*	*0*	*1*	*0*	*0.000*
FSIQ	*−0.088*	*0.04*	*6.08*	*0.01*	*0.92*	*0.853-0.982*
Fit index of this model: χ2 = 50.660 (p < 0.001), -2log likelihood = 48.262, discriminate accuracy = 96.9%						

note: B=unstandardized partial regression coefficient, SE = standard error, OR = odds ratio, Wald = (SE/B)2, CI = confidence interval.

dependent variable is existence of diagnosis ( 0 = no, 1 = childhood depression).

Independent variables: 5-HTTLPR genotype (0 = s/s, 1 = s/l, 2 = l/l), Adversity (maternal depression: 0 = no, 1 = yes). Gender (0 = female, 1 = male), SES(from 0 = low income to 5 = high income).

We conducted two-way ANOVA to examine the main and confounding effects on DSRS-C score in all participants. No significant G × E interaction between *5-HTTLPR* genotype and adversity or main effects of genotype, adversity, and gender was found from these analyses (Table 5). Additionally, the mean scores of DSRS-C in each genotype group were analyzed. No significant difference was found in any group.

**Table 5:** Two-way analysis of variance for investigating the main effect and G × E interaction

	**Type III sum of squares**	***F value***	***P value***
genotype (G)	*47.884*	*.302*	*.740*
adversity (E)	*7.175*	*.091*	*.764*
gender	*163.720*	*2.066*	*.154*
genotype (G) × adversity (E)	*117.151*	*.739*	*.480*
genotype (G) × gender	*82.514*	*.521*	*.596*
adversity (G) × gender	*2.451*	*.031*	*.861*

Dependent variable is Birleson score.

*R2* = 0.98 (adjusted *R2* = 0.10).

note: R = coefficient of determination, age and gender as covariates.

## Discussion

Interaction between adverse parental environment and gender showed a significant main effect despite a lack of G × E interaction in a Japanese pediatric sample. Because all participants in this study were ethnically homogenous, this is an important consideration for generalizing the present findings.

Depression is a critical and common condition found in children and adolescents as well as adults [3,38-40]. Early life stress is a risk factor for major depression, post-traumatic stress disorder (PTSD), and drug abuse, among other conditions [8,41]. In recent years, a possible association of the *5-HTTLPR* with youth depression has been traced in numerous studies [42-54]. Most studies were based on Caucasian populations, except for a few examining the Japanese general population [55,56]. No significant genotypic association of the *5-HTTLPR* polymorphism with depression in a Japanese pediatric population, particularly in patients who have adverse parental environment, was operationalized as the mothers’ history of recurrent major depressive disorder. This report is the first of genotypic association of the *5-HTTLPR* polymorphism in a Japanese pediatric population. Particularly, this study examined the interactive effect of *5-HTTLPR* and history of maternal depression among children and early adolescents with and without current depression. Moreover, few reports in the literature describe studies of G × E interaction in a sample of young people.

According to logistic regression analyses, our findings suggest that adversity (maternal depression), gender, and FSIQ had a main effect on current depression. Among them, adversity markedly affected the diagnosis of depression. However interpretation of the effect of FSIQ required careful consideration. A low score of FSIQ might be a cause and an effect [57]. More importantly, although G (genotype) × E interaction showed a significant main effect on the diagnosis of depression, it might explain the marked impact of maternal depression mediating the existence of diagnosis. Therefore, no interaction was found between the genotype and environmental factors in this case.

Additionally, no significant effect of G × E interaction was found with influences of adversity, gender, FSIQ, and age using multivariable logistic regression analyses. Our findings suggest that depression at an early age was mediated mainly by direct family adversity and gender. These results resemble findings reported previously in longitudinal studies [41,58,59].

Numerous behavioral genetics studies have yielded scientific knowledge related to interaction between genes and the environment [60]. Most importantly, interactive effects of genes and environmental factors are expected to appear cumulatively over a long period, suggesting that onset of depression results from long-term exposure to stressors. Therefore, an effect of G × E interaction might not be confirmed in this study because the sample comprised children and early adolescents. Our findings from models 1–3 and two-way ANOVA were in concordance with results from prior studies [23,61,62].

Meta-analyses of the *5-HTTLPR* G × E hypothesis have been described in many reports [17,22-24,63-65]. Sample age and gender composition emerge as important factors [64]. Furthermore, the S allele of *5-HTTLPR* has been implicated in the pathology of several neuropsychiatric phenotypes including mood and anxiety disorders [66,67] and in psychiatric disorders that involve serotonergic dysfunction [68,69]. Furthermore, we reported previously that sensitive periods exist, during which brain structures are most susceptible to exposure to early life events such as childhood maltreatment. These sensitive periods tend to correspond to times of rapid developmental change [70,71]. Therefore, we emphasize that continued monitoring is recommended for children who have early life stress as they pass through puberty. Reasons for the time lag between early life stress and depression are proposed along with potential strategies for early intervention [72].

Some limitations of this study and its results should be explained. First are the small number of participants and poor statistical power. Second, the covered environmental risk factors in the study were limited. Third, this was not a longitudinal study; no information related to outcomes in late adolescence is available for patients and HC groups. Fourth, the HC group significantly had high IQ than patient group, suggesting difficulty in case control. Fifth, female predominance was found in the patient group (Fisher exact, *P* = 0.008), which might influence the findings. Given these limitations, it will be important for future studies to ensure an adequate range of environmental variables and populations studied because a restricted range can strongly influence G × E results. Furthermore, to tease apart and thereby discern the respective influences of G × E, G × G, and E × E interactions, employing a molecular framework to extend this research would be beneficial. A follow-up study using both molecular genetics methods and measures of proximal environments is expected to provide a useful extension of the current study.

## Conclusions

In a Japanese pediatric sample, we examined whether adverse parental environment, operationalized as a mother’s history of recurrent major depressive disorder, interacts with *5-HTTLPR* polymorphism to predict a patient’s symptoms of depression. Data obtained from children and adolescents who had been carefully diagnosed categorically and data from age-matched controls were analyzed using logistic regression to assess G × E interaction. Despite an equivocal interaction effect, adversity and gender showed a significant main effect.

## Abbreviations

(G × E): Gene-by-environment; (5-HTTLPR): Serotonin-transporter-linked polymorphic region; (S): Short allele; (L): Long allele; (DSM-IV-TR): Diagnostic and Statistical Manual of Mental Disorders, Fourth Edition, Text Revision; (SD): Standard deviation; (SCID): Structured Clinical Interview for DSM-IV; (HC): Healthy controls; (ADHD): Attention deficit hyperactivity disorder; (SDS): Zung self-rating depression scale; (DSRS-C): Birleson Depression Self-Rating Scale for Children; (WISC-III): Wechsler Intelligence Scale for Children – Third Edition; (CBCL-YSR): Child Behavior Checklist Youth Self-Report; (SES): Socioeconomic status; (PCR): Polymerase chain reaction; (ANCOVA): Analysis of covariance; (ANOVA): Analysis of variance; (FSIQ): Full-scale IQ; (VIQ): Verbal IQ; (PIQ): Performance IQ; (PTSD): Post-traumatic stress disorder.

## Competing interests

The authors reported no biomedical financial interests or potential conflicts of interest. The authors have declared that no competing interests exist.

## Authors’ contributions

All authors have contributed to the manuscript as described below: AT designed and supervised the whole study, and AT and JK recruited and assessed the eligible patients with interviews and neuropsychological evaluation. TT and MO collected healthy control blood samples. SN, TXF, and KS conducted genotyping. Statistical analyses were conducted by AT and NM. The manuscript was originally written by AT and was revised by NM. However, all authors assisted in the revision process. All authors read and approved the final manuscript.

## Pre-publication history

The pre-publication history for this paper can be accessed here:

http://www.biomedcentral.com/1471-244X/13/134/prepub
